# Untangling neurodevelopmental disorders in the adulthood: a movement disorder is the clue

**DOI:** 10.1186/s13023-022-02218-8

**Published:** 2022-02-16

**Authors:** Elisabetta Indelicato, Michael Zech, Matthias Amprosi, Sylvia Boesch

**Affiliations:** 1 Center for Rare Movement Disorders Innsbruck, Medical University of Innsbruck, Anichstrasse 35, 6020 Innsbruck, Austria; 2 Institut for Neurogenetics, Helmholtz Zentrum München, Ingolstädter Landstraße 1, 85764 Oberschleißheim, Munich-Neuherberg, Germany; 3 Institute of Human Genetics, Technical University of Munich, Munich, Germany

**Keywords:** Neurodevelopmental disease, Developmental delay, Dystonia, Tremor, Genetic diagnosis

## Abstract

**Background:**

The genetic landscape of neurodevelopmental disorders is constantly expanding and children with early-onset neurological phenotypes increasingly receive a genetic diagnosis. Nonetheless, the awareness of the chronic course of these conditions, and consequently their recognition and management in the adult population, is still limited.

**Results:**

Herein, we describe four patients with rare neurodevelopmental disorders (*SON*, *ZMYND11, DNMT1* and *YY1-*related diseases), who received a genetic assignment only in the adulthood. All these patients had an early developmental delay and displayed a movement disorder (dystonia/ataxia/tremor) which manifested for the first time, or worsened, in the adulthood, prompting the referral to a neurologist. This phenotypic combination led eventually to the genetic testing. We report previously unrecognized features and highlight the peculiarities of the adult presentation of four neurodevelopmental disorders.

**Conclusions:**

This report expands the current knowledge on four rare neurodevelopmental disorders (*SON*, *ZMYND11, DNMT1* and *YY1*), which was mainly based on reports from paediatric cases. This case series emphasize the importance of a tight neurological surveillance extending beyond the childhood.

## Introduction

In the last decade, the development of high-throughput sequencing techniques unraveled the previously unrecognized genetic background of syndromes with neurodevelopmental features and intellectual disability [1]. Consequently, also the genetic basis of disorders, which were traditionally attributed to so-called symptomatic causes, such as cerebral palsy or epileptic encephalopathies, is constantly increasing [2].

In the field of neurodevelopmental disorders, clinical research is traditionally focused on the pediatric population [3]. Despite the recent advances, the diagnosis and management of adult patients with these conditions still represents an unfilled gap. Adults with neurodevelopmental conditions are unlikely to receive a genetic diagnosis, notwithstanding the great value that their evaluation would add to our understanding of the evolution of these disorders.

In the present vignette series, we present four cases of patients with neurodevelopmental conditions, which received a genetic diagnosis only in the adulthood. In all these cases, the occurrence of a disabling progressive movement disorder initiated the reevaluation process. Eventually the syndromic nature of the observed phenotype gave the hint for the disposal of a genetic testing.

## Methods

**Fig. 1:**
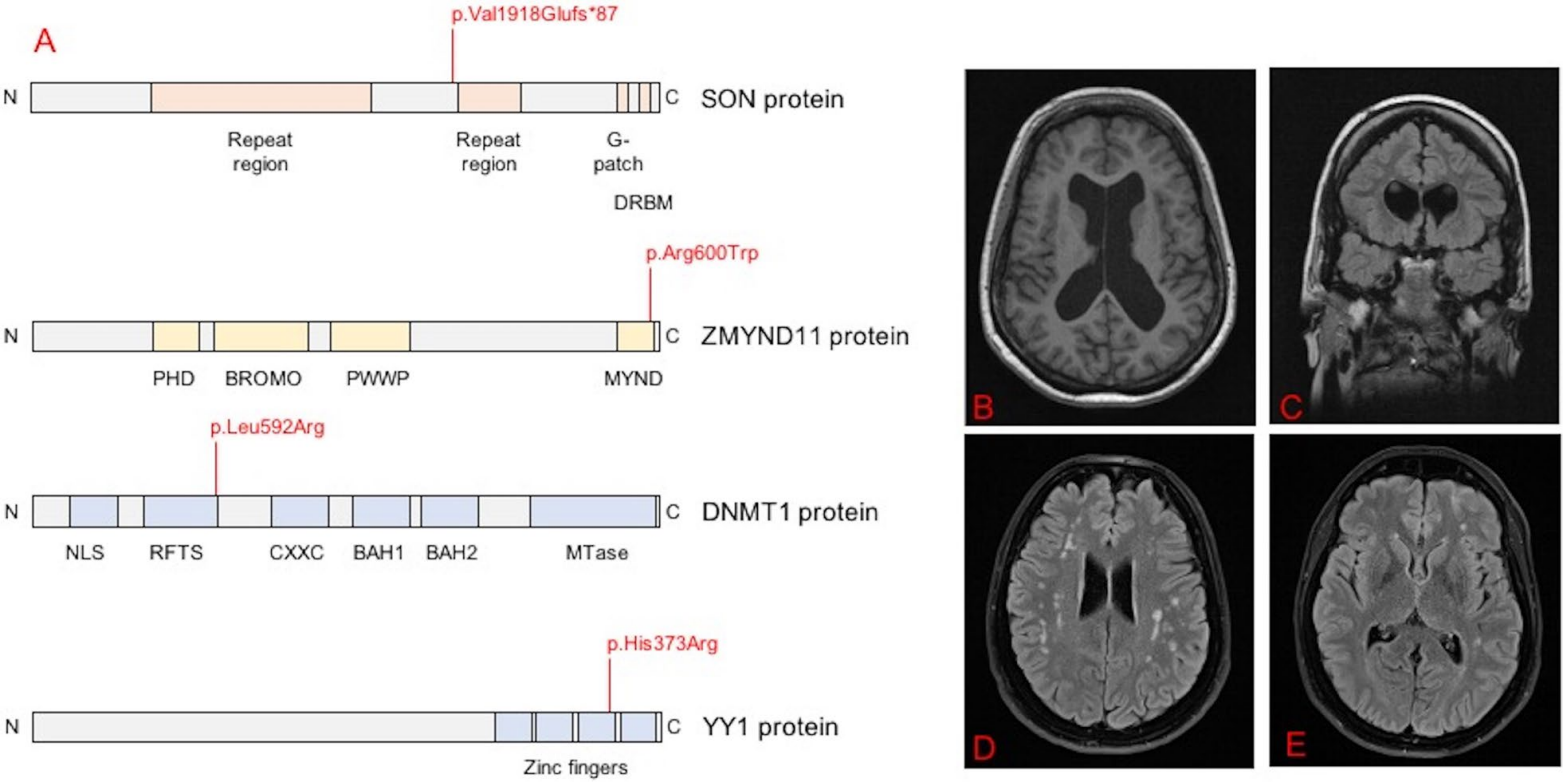
Detected mutations in *SON*, *ZMYND11*, *DNMT1* and *YY1* and related brain imaging features. **a** Protein changes related to the described mutation in *SON*, *ZMYND11*, *DNMT1* and *YY1*. **b**, **c** 1,5 Tesla brain MRI-Imaging from patient 2 (*ZMYND11*-related disease) showing global atrophy as well as turricephaly; **b** T1-axial sequence, **c** T2 dark-fluid coronal sequence. **d, e** 1,5 Tesla brain MRI-Imaging from patient 3 (*YY1*-related disease) showing a normal appearance of basal ganglia and multiple unspecific white matter lesions; T2 dark-fluid axial sequences

This study was approved by the local ethical committee and written informed consent was obtained from all participating patients or their legal representatives as appropriate. The cases were identified within the frame of an ongoing genomic research study of dystonic disorders. Genetic screening and variant detection were performed as previously reported [4]. In short, whole exome sequencing (WES) was performed on genomic DNA from the cases presented in this study. In solution exome capture, sequencing, bioinformatics, and variant prioritization by population frequency, predicted effect on the protein level, deleteriousness annotation, and known pathological effect were carried out as previously described [4, 5]. Cases with variants in *ZMYND11* and *DNMT1* were analysed using a parent-affected child trio design, whereas cases with variants in *SON* and *YY1* were analysed as singletons. Sanger sequencing was used to verify all prioritized variants and test for co-segregation. The variant findings presented herein (Fig. 1) were mentioned previously but without detailed clinical information of the carrier patients [4, 5].

## Case descriptions

### Patient 1: SON-related disease

This patient was seen for the first time at the age of 39, when he was referred to our outpatient clinic because of an increasing disabling tremor of the extremities.

The patient presented a severe intellectual disability; he was living in an institution for people with disabilities and the clinical history had to be collected through his mother. In the neurological examination, he showed a cervical dystonia, a marked action tremor, more pronounced in the right arm, as well as dystonic jerks of the extremities.

Concerning the earlier clinical history, the mother reported that, after an unremarkable pregnancy and delivery, a sucking weakness manifested already in the first day after birth. Subsequently, a delay in the acquisition of the milestone became evident (sitting with 10 months, walking with 18 months). He did show some dysmorphic features consisting of deep-set eyes with broad nasal bridge and low-set ears. When he was 3 years old, he developed febrile convulsions, which were successfully treated with phenobarbital. Later on, at the age of 9, after a trivial head trauma, he acutely developed a coma state with high fever, which was initially interpreted as an encephalitis and empirically treated with acyclovir. He progressively recovered within days. According to the mother, the performed evaluation of the cerebrospinal fluid yielded no abnormalities. Cumulatively, he developed four times similar symptoms, with acute onset of a coma state and fever, mostly after trivial trauma. The later episodes were also accompanied by hemiparesis and headache. Notably, he suffered from migraine until the age of 19. The family history was unremarkable.

A brain MRI at the age of 31 revealed a posttraumatic/peripartal gliosis in the right parietooccipital region, otherwise no further relevant findings.

Singleton WES identified a heterozygous frameshift variant in SON (c.5753_5756del, p.Val1918Glufs*87). Sanger evaluation did not detected this variant in the parents, demonstrating its de novo occurrence.

### SON: gene function, related phenotype and peculiarities of the present case

*SON* encodes a RNA-binding protein that localizes at the nuclear speckles and acts as a mRNA splicing cofactor [6]. The gene *SON* was related for the first time to human disease in 2016 in a WES cohort including mostly pediatric patients with intellectual disability and developmental delay [7]. In this first description and in following reports, affected individuals carry de novo mutations, mostly truncating. The patronymic Zhu-Tokita-Takenouchi-Kim syndrome (or ZTTK syndrome) was coined for this condition.

Beyond the cardinal developmental delay, a majority of patients display further features including hypotonia in infancy, seizures, autisms, dysmorphic facies and various brain malformation [7–9]. Early feeding difficulties are an almost universal feature. Up to date, three adult patients out of 28 described cases have been reported [7]. The mutation identified in our case has been recurrently described [7], though, to the best of our knowledge, no association with movement disorders or hemiplegic migraine has been described to date.

### Patient 2: ZMYND 11-related disease

The patient 2 presented at our centre at the age of 34. At the time of referral, the general examination showed craniofacial dysmorphic features with hypertelorism and micro/turricephaly, slight scoliosis and genu valgus. The neurological phenotype encompassed a severe mental retardation, therapy-resistant seizures, marked dyslalia as well as a broadened basis ataxic gait with dystonic position of the left hand. She was on five anticonvulsive drugs and under Vagus-nerve stimulation.

**Fig. 2:**
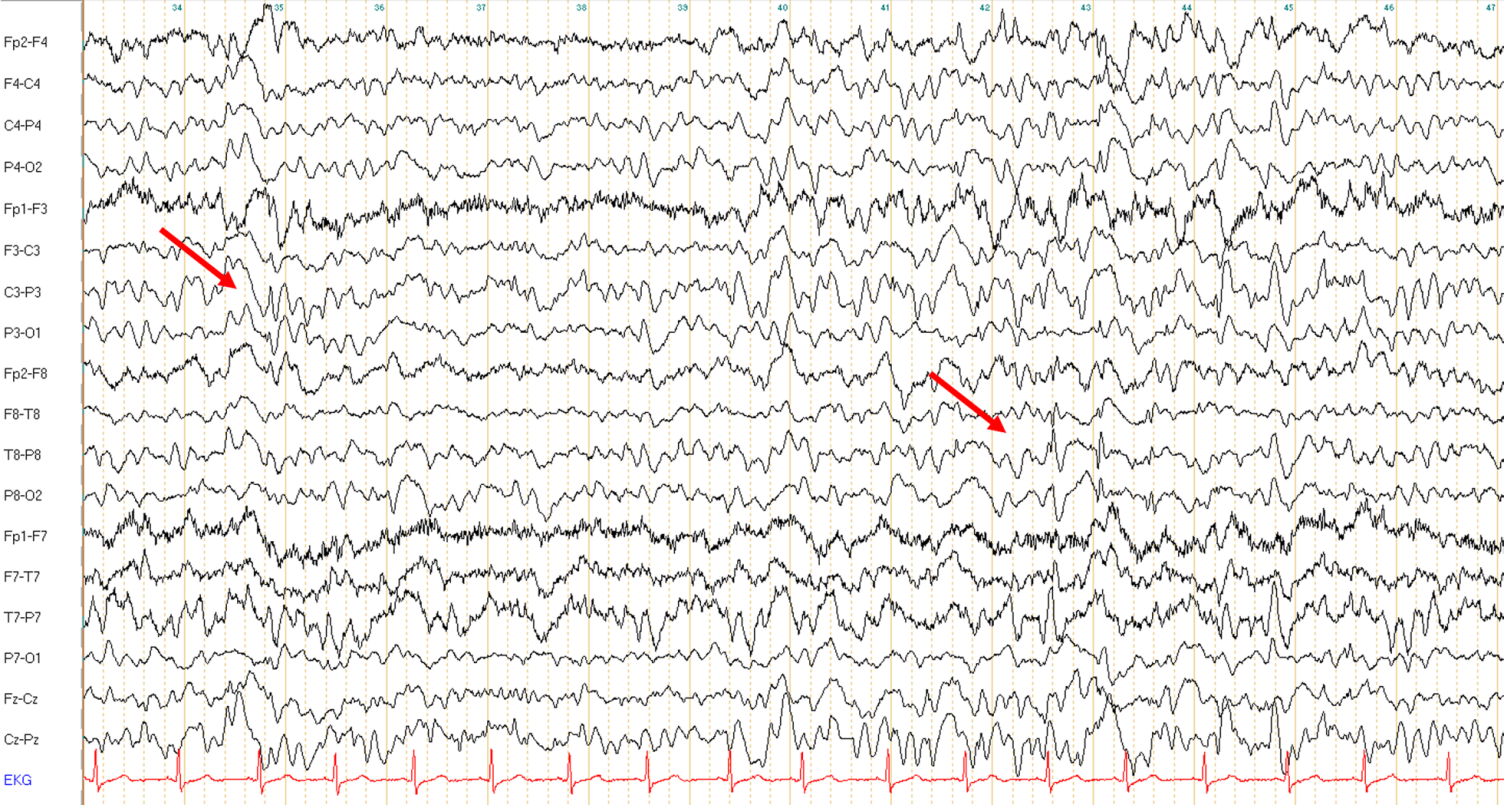
EEG recording in an adult patient with *ZMYND11* disease. Routine scalp-EEG recording from patient 2 at the age of 34: Generalized slowing in theta-delta frequencies as well as multifocal spikes (arrows) are evident. Bipolar longitudinal montage with 70 Hz filter and time constant of 0.3 s; sensitivity 7 μV

The patient was the first of three children. According to the mother, already during the pregnancy a delayed growth of the foetus was noticed as well as a placenta dysfunction, the delivery was prolonged. After birth, a delayed growth as well as a delay in the acquisition of the milestones became evident. She started speaking with 18 months and started walking at 3 years of age. At the age of 4, she had her first seizure and at the age of 8 a second one occurred. From the age of 20, seizures started again and became the most troublesome symptoms in the following years. She indeed developed multiple, therapy resistant seizures (myoclonic, grand-mal) as well as atonic seizures starting from the age of 24. While the first EEG examinations in the childhood yielded unremarkable findings, later in the adulthood, a marked bilateral slowing as well as multifocal polyspikes and slow-spike-waves became evident (see Fig. 2). The brain imaging at the age of 26 showed a global atrophy. Trio WES identified a heterozygous de novo missense variant in *ZMYND1* (c.1798C > T, p.Arg600Trp).

### ZMYND 11: gene function, related phenotype and peculiarities of the present case

*ZMYND11* is a tumour suppressor gene coding for a histone-binding protein [10]. By inhibiting the elongation mediated by the RNA polymerase II it critically represses the transcription of gene subsets, which are essential for tumour cell growth. Furthermore, *ZMYND11* appears to have an inhibitory role in the neuronal differentiation. *ZMYND11* was initially identified as candidate gene in the setting of an intellectual disability syndrome associated with 10p15.3 deletion [11]. This links was corroborated by the description of eight patients with developmental delay and both de novo and inherited truncating *ZMYND11* mutations in 2014 [12]. Up to date, around 26 patients, of which six adults, have been reported in the literature. Intellectual disability is mild to severe and particularly the speech skills are affected. Behavioural disturbances spanning from autism spectrum disorders to attention-deficiency hyperactivity disorders represent a further cardinal feature. Epilepsy belongs to the phenotype in 40% of patients [13]. Concerning imaging features, two reports described delayed myelination and cerebral atrophy [14, 15]. Heterozygous truncating mutations are the classical finding, while up to date missense mutations have been described in only two cases [11, 14], one of whom bearing the same variant of the present case. In this child, dysmorphic features as well as the severe early phenotype with intrauterine growth retardation are fairly similar to our case. Conversely, most of the adult cases reported to date harboured a milder phenotype, which did not impair the reproductive fitness. Up to date, ataxia and dystonia were described in one patient and two further patients respectively [12].

### Patient 3: DNMT1-related disease

This patient was referred at the age of 42 because of a disabling tremor of the head and extremities. At this time, he showed an ataxic syndrome with dysarthria, dysmetria of extremities and widened based gait, a dystonic head tremor with antero- and laterocollis and an evident geste antagoniste, as well as brisk tendon reflexes in the legs. Furthermore, he was affected by sensorineural hearing loss. No obvious dysmorphic features were evident at the examination.

The patient was born after an uneventful pregnancy and delivery. The neonatal developmental was unremarkable. Later, a slight delay in the acquisition of speaking skills became evident. With 4–5 years of age, he underwent the first ENT examination because of suspicion of hearing loss; this was unremarkable. Because of the delay in speaking, he started the primary school one year later. Concerning motor skills, the parents described a rather clumsy child. Starting from the puberty, he developed a tremor, as well as problems with balance. In the school, he had a normal performance and obtained its diploma from a commercial college. From the age of 34, he has hearing aiding devices. The family history was unremarkable.

In the brain MRI at the time of referral a global brain volume loss was evident und more pronounced in the posterior fossa. The electrophysiological examination showed a sensorimotor axonal neuropathy. In the neuropsychological testing an impairment in speaking, comprehension, as well as a reduced IQ were noticed, for which a major contribution of the hearing loss was assumed. Trio WES detected a heterozygous de novo missense variant in *DNMT1* (c.1775 T > G, p.Leu592Arg), which was not described previously.

### DNMT1: gene function, related phenotype and peculiarities of the present case

*DNMT1* codes for the key maintenance DNA-methyltransferase in mammalian cells and thus plays a pivotal role in the transcriptional repression [16]. Ten years ago, heterozygous mutations in the *DNMT1* gene were described for the first time in the setting of variable neurological phenotypes encompassing sensory neuropathy, cerebellar ataxia, deafness, narcolepsy and cognitive disturbances [17–19]. The mutations occurred both in families with an autosomal dominant inheritance pattern and de novo. In the larger series reported to date, the most frequent presenting symptom was sensorineural deafness, which was invariably followed by sensory neuropathy leading to ulcerations, arthropathy as well as contributing to gait ataxia [17]. The median age at onset was 37, with a presentation in the first two decades occurring rarely in the setting of de novo mutations [17, 20]. All mutations reported to date, including the newly described one in our case, are missense and reside in the TS-domain, which controls the binding to hemimethylated cytosines, the preferred substrate of *DNMT1* [21]. Comparing to the other cases in the literature, the combined movement disorder consisting of disabling tremor and dystonia was the most prominent and disabling feature in our patient. The early begin also distinguishes it from the usual phenotype. After detection of the causal *DNMT1* mutation, we revaluated our patient and elicited symptoms suggestive of narcolepsy, which was then confirmed in a polysomnographic examination. Hypocretin levels in cerebrospinal fluid were in the normal range. Because of the therapy resistant tremor, evaluation concerning deep brain stimulation is ongoing.

### Patient 4: YY1-related disease

This patient was referred by her family doctor at the age of 46 because of a tremor. At the general examination, a mild facial dysmorphia with low set ears was evident. The neurological examination revealed a parkinsonian syndrome with slight hypomimia, hypophonia as well as bradykinesia of the left hand at finger-tapping, as well as dystonic features, consisting of graphospasm on the right and dystonic inversion of the left leg with intermittent dystonic tremor. Moreover, the patient suffered from hearing problems, which at the ENT examination were consistent with sensorineural hearing loss.

Detailed information about early clinical history were not available. According to the relatives, she had marked learning difficulties as a child and repeated classes. The graphospasm was present at least since the school age. The current occupation as waitress was possible only within the family enterprise. The family history was unremarkable.

At the time of referral, the brain MRI showed several unspecific gliotic changes, no further pathology. The dopamine transporter imaging was unremarkable. Singleton WES revealed a likely pathogenic heterozygous missense mutation in YY1 (c.1118A > G, p.His373Arg). Sanger sequencing showed that the variant was not present in the patient`s healthy mother and brother.

### YY1: gene function, related phenotype and peculiarities of the present case

Yin Yiang 1 (*YY1*) is a ubiquitous transcription factor, which regulates manifold processes underlying embryogenesis, cellular growth and differentiation [22]. Heterozygous pathogenic variant or heterozygous deletion of the *YY1* containing locus 14q32.2 underlie a clinical phenotype characterized by developmental delay and intellectual disability, which has been named “Gabriele-de Vries syndrome” [23]. Up to date, less than 20 patients with this condition have been reported in the literature. While the majority of them are children with a syndromic intellectual disability, the few adult patients showed a progressive dystonia as prominent feature [23, 24]. Also, in our case, a progression of the movement disorder became evident in the later follow-up and caused increasing gait difficulties.

## Discussion

In the last decade, significant advancements in the field of medical genetics led to the increasing recognition of genetic aetiologies of neurological phenotypes with early onset [1]. Nonetheless, the awareness of the chronic nature of neurodevelopmental disorders and of their possible evolution in the adulthood is still poor [3]. Herein, we exemplified this issue by describing the diagnostic crusade of four patients. In all these cases, the reappraisal of the disease aetiology followed a neurological revaluation in their 30-40ies, considering the syndromic clinical picture and the global course of the disease. All four patients exhibited some early developmental features, namely a delay in cognitive and/or motor milestones, but displayed their distinct movement disorder phenotype only in the adulthood. Three of the diagnosed conditions (*SON*, *ZMYND 11,* and *YY1* diseases) were originally recognized in children and clinical descriptions of adult cases in the literature are exceptional. On the contrary, *DNMT1* related disorders typically manifest in adults and only recent reports highlighted how de novo mutations, as in our case, may result in an earlier onset. Concerning the related phenotype, we described herein for the first time dystonia and hemiplegic migraine as additional features of *SON*-related disease.

Over than a century ago, the term “cerebral palsy” (CP) was coined to describe neurodevelopmental phenotypes primarily affecting movement and posture [2]. Since then, the diagnosis of CP has been traditionally applied to describe the chronic neurological sequelae of various perinatal noxae such as intrauterine infections, hypoxia, birth trauma. Nonetheless, a subset of patients with CP-like phenotypes is known to bear a genetic condition and with the increasing improvement of genetic tools, this group is constantly expanding [2]. Consequently, our clinical approach to these disorders needs to be refined, in order to optimize their recognition and management. With this objective, international joined efforts eventually translated in the development of dedicated clinical networks such as the ERN RND [25].

In the early evaluation of a patient with a neurodevelopmental phenotype, several clinical features may hint at a genetic aetiology: the lack of perinatal risk factor, missing imaging findings of brain injury or a motor symptoms onset after an initial period of normal development [2]. In several cases, only the clinical evolution with onset of a classical movement disorder phenotype such a prominent ataxia or dystonia beyond the childhood may give the decisive hint. If milder neurodevelopmental features did not raise attention in the childhood, the adult neurologist may be the first specialist in charge of untangling a possible neurodevelopmental disorder. In adults, recalling delayed milestones may be challenging. Evidence from genetic studies on dystonia shows however that an onset < 21 years of age along with a syndromic phenotype with > 1 neurological feature predicts a high diagnostic yield of WES [5]. These considerations underline the importance of an ongoing deep phenotyping in the frame of a regular neurological surveillance [3].

Achieving a genetic diagnosis bears relevant consequences for life planning for the patients and their relatives, allows a better evaluation of disease prognosis and treatment strategies. For example, the underlying genotype can predict a response to the treatment with deep brain stimulation in genetic dystonia. Very recently, a positive effect of this treatment was reported also in *YY1-*disease [26]. Eventually, an early genetic assignment and diagnosis will possibly accelerate the access to causative therapy in the future.

## Data Availability

Detailed WES data are available upon request.
